# Supplementary material for: Ferritin-mediated iron detoxification promotes hypothermia survival in Caenorhabditis elegans and murine neurons
Source: Nat Commun. 2022 Aug 19;13:4883. doi: 10.1038/s41467-022-32500-z (PMC9391379; doi:10.1038/s41467-022-32500-z)
Supplement: Supplementary file 1 — Supplementary information [file 41467_2022_32500_MOESM1_ESM.pdf]

SUPPLEMENTARY INFORMATION

Title: Ferritin-mediated iron detoxification promotes hypothermia survival in *Caenorhabditis elegans* and murine neurons

Authors: Tina Pekec, Jarosław Lewandowski, Alicja A. Komur, Daria Sobańska, Yanwu Guo, Karolina Świtońska-Kurkowska, Jędrzej M. Małecki, Abhishek Anil Dubey, Wojciech Pokrzywa, Marcin Frankowski, Maciej Figiel, and Rafal Ciosk

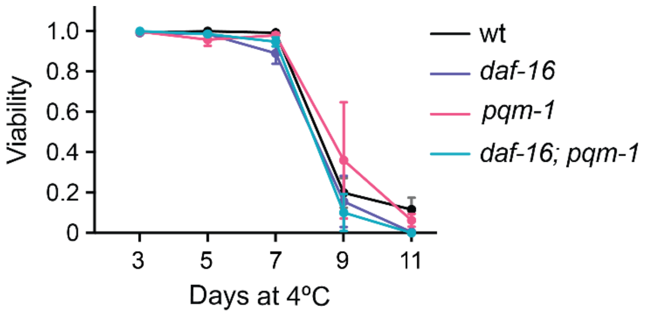

Figure S1. Inactivation of *daf-16* and *pqm-1* in wt animals has no effect on cold survival.

Relevant to Fig. 2

Animals of the indicated genotypes were subjected to cold as in Fig. 1a. Tested strains were: wt, *daf-16(mu86)*, *pqm-1(ok485)*, and *daf-16(mu86); pqm-1(ok485)* double mutant. There was no obvious difference in cold survival between any of the examined animals. Error bars represent SEM, n= 3 independent experiments. 200-300 animals were scored per time point. Source data are provided as a Source Data file.

a

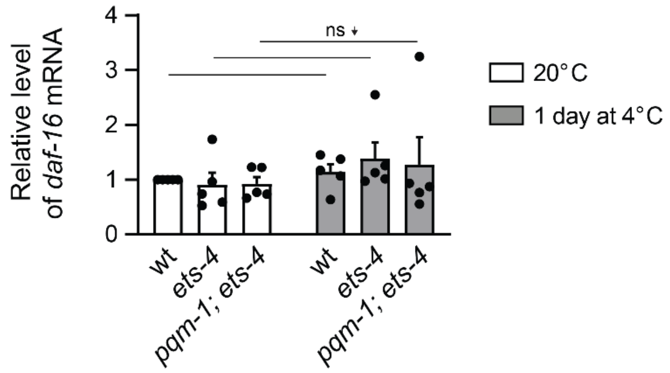

b

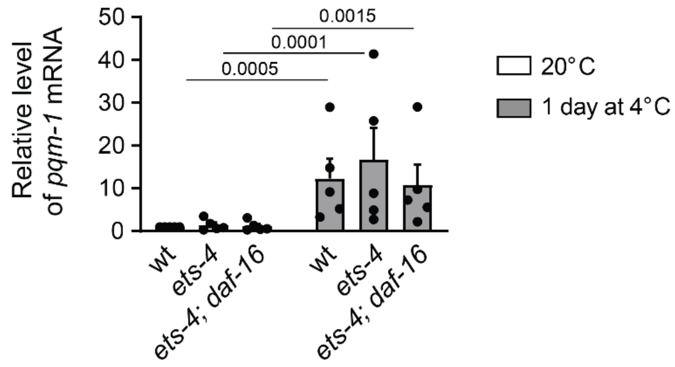

Figure S2. Evaluation of *daf-16* and *pqm-1* mRNAs levels in the cold.

Relevant to Fig. 3

a. Animals (wild type (wt), *ets-4(rrr16)* and *pqm-1(ok485)*; *ets-4(rrr16)* double mutants) were collected at 20°C, and after 1 day at 4°C, as in Fig. 1a. The level of *daf-16* mRNA, measured by RT-qPCR, was normalized to *act-1* mRNA, and is shown relative to the *daf-16* mRNA level in wt at 20ºC. Error bars represent SEM, n= 5 independent experiments. ns, not significant (2-way ANOVA with Sidak's multiple comparison test).

b. Animals (wild type (wt), *ets-4(rrr16)* and *daf-16(mu86)*; *ets-4(rrr16)* double mutants) were collected at 20°C, and after one day at 4°C, as in Fig. 1a. The level of *pqm-1* mRNA, measured by RT-qPCR, was normalized to *act-1* mRNA, and is shown relative to the *pqm-1* mRNA level in wt at 20ºC. Error bars represent SEM, n= 5 independent experiments. *p* values calculated using 2-way ANOVA with Sidak's multiple comparison test.

Source data are provided as a Source Data file.

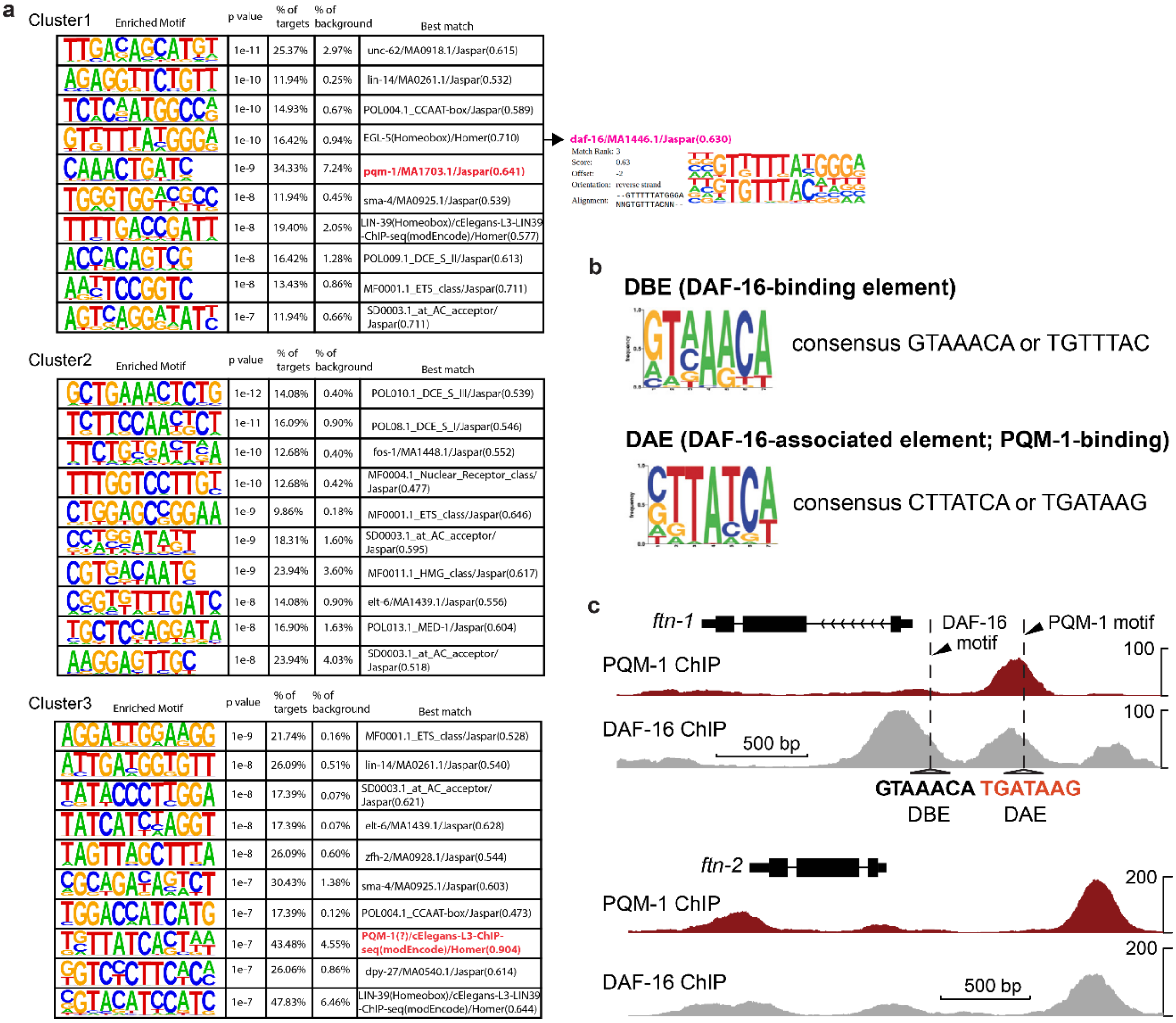

Figure S3. Identification of a candidate gene potentially promoting cold survival.

Relevant to Figs. 4 and 5

a. De novo motif enrichment analysis of each gene cluster in Fig. 4a. The enrichment analysis was performed with HOMER [1], for each cluster with the following parameters: -start -1500, -end 1500, -p 6. All genes in the genome were used as background. Only the top 10 enriched motifs per gene cluster are shown. The DAF-16 binding motif in Cluster 1 analysis was ranked 3rd within the group of EGL-5-like motifs.

b. DBE (*D*AF-16-*b*inding *e*lement) and DAE (*D*AF-16-*a*ssociated *e*lement) from Tepper *et al.*[2]

c. Distribution of DAF-16 and PQM-1 ChIP peaks over the *ftn-1* and *ftn-2* genomic regions. Thick and thin bars indicate, respectively, exons and untranslated regions, and lines introns. Approximate positions of DBE and DAE motifs, with DNA sequences indicated, are shown over the *ftn-1* region. Based on the modENCODE data.

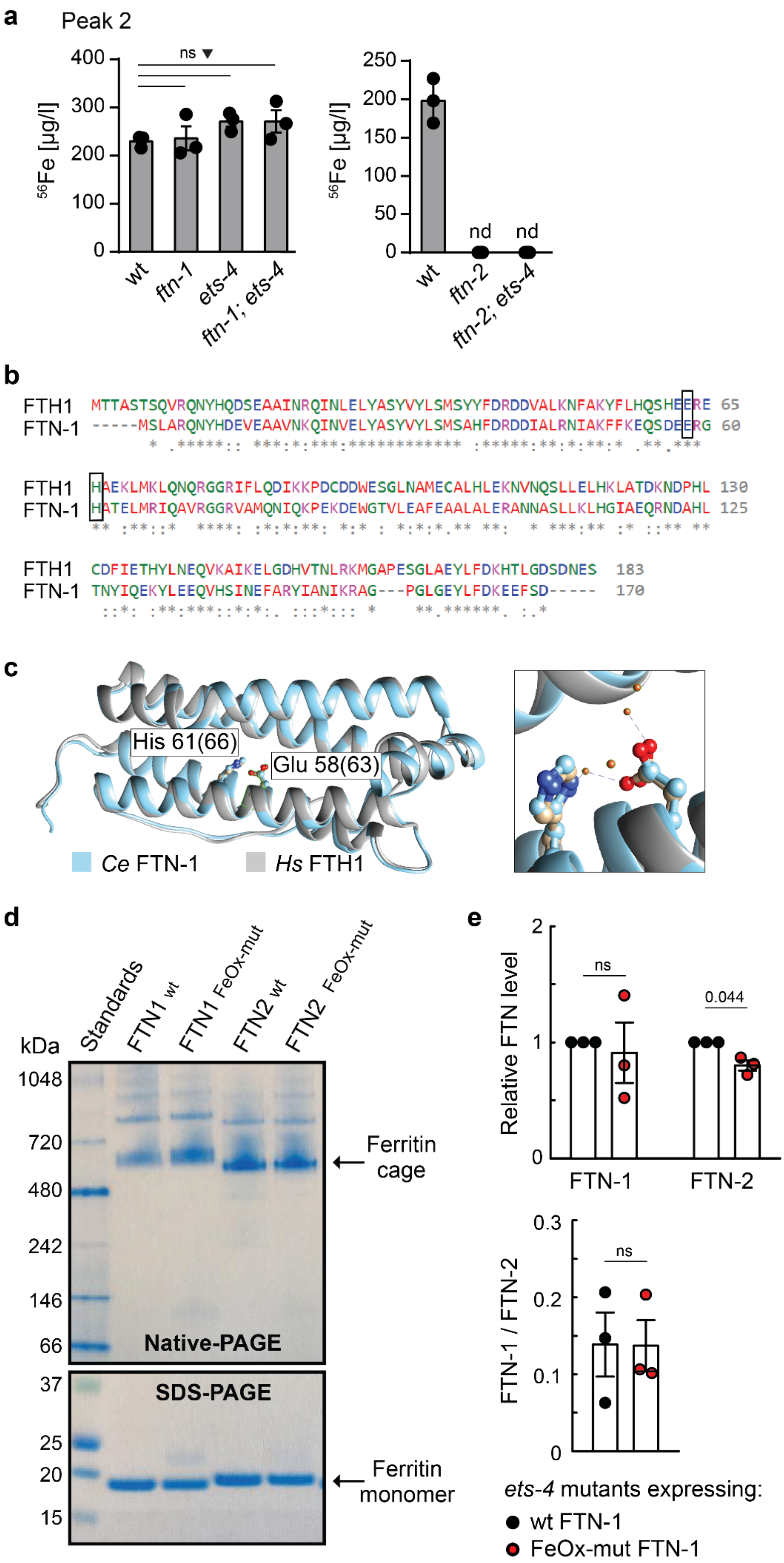
a
Peak 2
56Fe [µg/l]
400
300
200
100
0
ns ▼
wt
ftn-1
ets-4
ftn-1; ets-4
250
200
150
100
50
0
nd
nd
wt
ftn-2
ftn-2; ets-4
b
FTH1 MTTASTSQVRQNYHQDSEAAINRQINLELYASYVYLSMSYYFDRDDVALKNFAKYFLHQSHEERE 65
FTN-1 -----MSLARQNYHDEVEAAVNKQINVELYASYVYLSMSAHFDRDDIALRNIAKFFKEQSDEERG 60
FTH1 HAEKLMKLQNQRGGRIFLQDIKKPDCDDWESGLNAMECALHLEKNVNQSLLELHKLATDKNDPHL 130
FTN-1 HATELMRIQAVRGGRVAMQNIQKPEKDEWGTVLEAFEAALALERANNASLLKLHGIAEQRNDAHL 125
FTH1 CDFIETHYLNEQVKAIKELGDHVTNLRKMGAPESGLAEYLFDKHTLGDSDNES 183
FTN-1 TNYIQEKYLEEQVHSINEFARYIANIKRAG---PGLGEYLFDKEEFSD----- 170
c
His 61(66)
Glu 58(63)
Ce FTN-1
Hs FTH1
d
kDa
Standards
FTN1 wt
FTN1 FeOx-mut
FTN2 wt
FTN2 FeOx-mut
1048
720
480
242
146
66
Native-PAGE
Ferritin cage
37
25
20
15
SDS-PAGE
Ferritin monomer
e
Relative FTN level
2
1
0
ns
0.044
FTN-1
FTN-2
FTN-1 / FTN-2
0.3
0.2
0.1
0
ns
ets-4 mutants expressing:
wt FTN-1
FeOx-mut FTN-1

Figure S4. FTN-1 promotes cold survival *via* its ferroxidase activity.

Relevant to Fig. 6

a. Quantitation of ferritin-associated iron (Peak 2) in Figs. 6a and b, calculated relative to ferritin standard. Only loss of *ftn-2* decreased ferritin-associated iron. Strains used: wild type (wt), *ets-4(rrr16)*, *ftn-1(ok3625)*, *ftn-1(ok3625); ets-4(rrr16)*, *ftn-2(ok404)*, *ftn-2(ok404)*; *ets-4(rrr16)*. Error bars represent SEM, n= 3 independent experiments. ns, not significant (unpaired two-sided t-test). nd, not detected.

b. Amino acid sequence alignment of *H. sapiens* ferritin heavy chain 1 (FTH1; NCBI accession number NP_002023.2) and *C. elegans* ferritin (FTN-1: NCBI accession number NP_504944.2). The alignment was based on the multiple sequence alignment software Clustal Omega [3]. Residues mutated in *ftn-1*, yielding a ferroxidase-inactive protein, are boxed. Asterisks (*) indicate fully conserved residues, colons (:) residues with strongly similar properties, and periods (.) residues with weakly similar properties.

c. Structural alignment of *H. sapiens* ferritin heavy chain 1 (FTH1 – colored in grey, PDB code 4OYN) and *C. elegans* FTN-1 (colored in light blue), using the Phyre2 tool [4]. Amino acids critical for the ferroxidase activity are shown as balls and sticks. The colors indicate: carbon atoms in FTH1 (light brown) or FTN-1 (light blue), oxygen atom of glutamic acid (red), and nitrogen atom of histidine (dark blue). The magnification shows the ferroxidase active site, with the iron atoms shown as dark orange balls and coordination bonds as dotted lines.

d. Recombinant FTNs form high molecular weight complexes *in vitro*. Tag-less FTN-1 or -2, either WT or E58K/H61G-mutated (FeOx-mut), were resolved in non-denaturing conditions by Native-PAGE (top, 2 µg/lane) or by denaturing SDS-PAGE (bottom, 1 µg/lane), in the presence of appropriate molecular weight standard. Gels were stained with Coomassie Blue. Shown is a representative image, out of two independent experiments, obtained with similar results. Arrows indicate position of ferritin cage (top) and monomer (bottom). The uncropped scan of gels are presented at the end of this Supplementary Information file.

e. Similar expression of wt and FeOx-mut FTN-1 in *ets-4(-)* mutants. The *ets-4(rrr16)* animals, expressing either wt or FeOX-mut FTN-1 (*ftn-1(syb2550)*), were cold-exposed for 3 days, as in Fig. 1a. The FTN-enriched extracts were prepared and analyzed by mass spectrometry for the presence of FTN-1 and FTN-2. Top: integrated signal from FTN-1- and FTN-2-specific peptides, normalized to background proteins, shown relative to the *ets-4(-)* values. Bottom: the ratio of either wt or FeOx-mut FTN-1 to FTN-2. Error bars represent SEM, n= 3 independent experiments. *p* values calculated using paired two-sided t-test; ns, not significant. Source data are provided as a Source Data file.

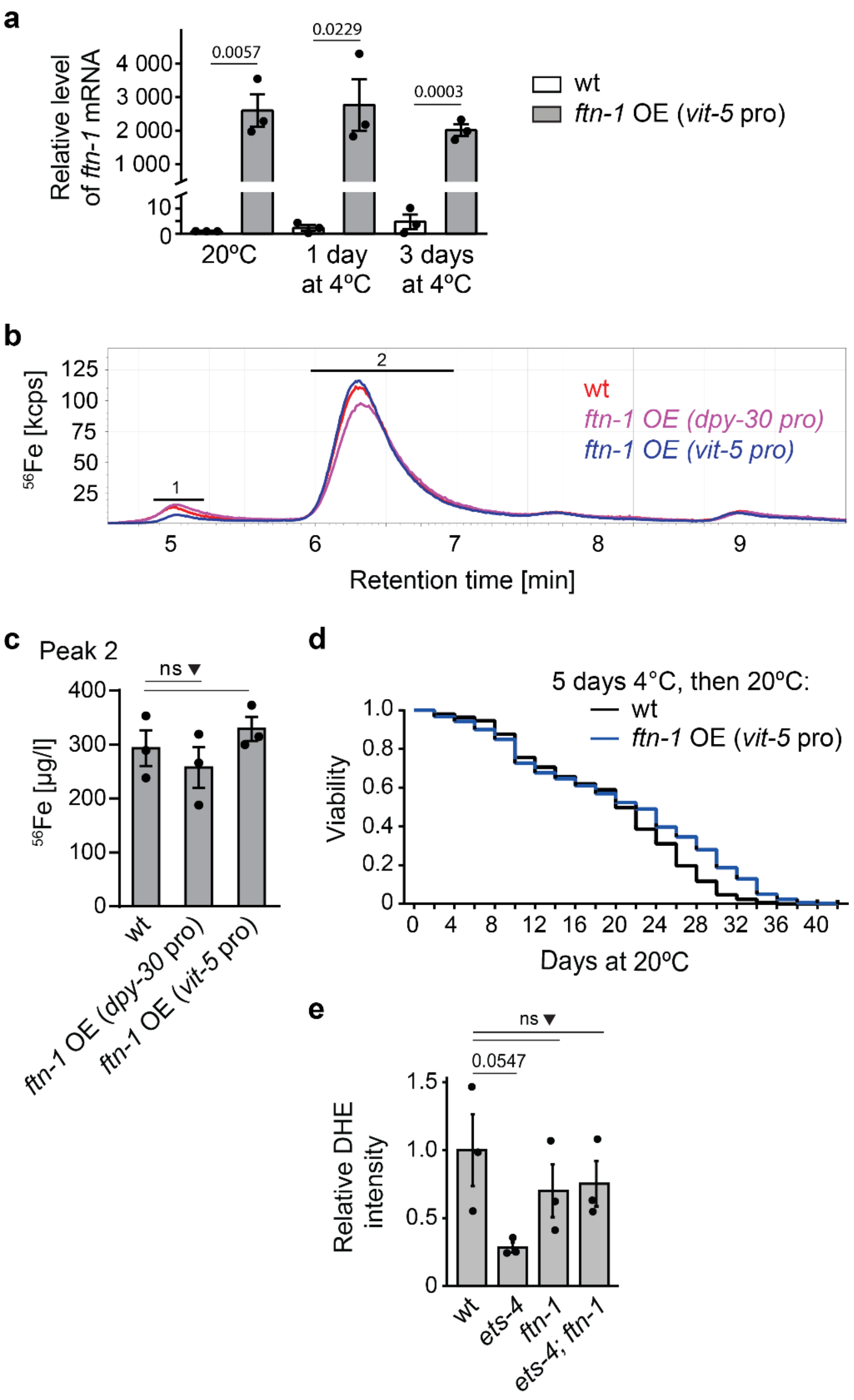

Figure S5. FTN-1 overexpression is sufficient for enhanced cold survival.

Relevant to Fig. 7

a. The level of *ftn-1* mRNA, measured by RT-qPCR, in animals of the indicated genotypes. 1-day-old adults were collected at 20°C or after one and three days at 4°C. The mRNA level was normalized to *act-1* mRNA. At each time point, the values were then normalized to the wt

at 20°C. Note greatly elevated level of *ftn-1* in the strain overexpressing it (OE) from the *vit-5* promoter (*vit-5* pro). Error bars represent SEM, n= 3 independent experiments. *p* values calculated using unpaired two-sided t-test.

b. Native soluble iron-binding species separated and detected by SEC-ICP-MS like in Figs. 6a and b. Iron is mostly associated with high molecular weight complexes (Peak 1) and ferritin (Peak 2). Note that FTN-1 overexpression (from either *vit-5* or *dpy-30* promoter) contributes little or nothing to stored iron.

c. Quantitation of ferritin-associated iron (Peak 2) in **b**, calculated relative to ferritin standard. FTN-1 overexpression did not have any obvious effect on iron within Peak 2 (ferritin-associated). Error bars represent SEM, n= 3 independent experiments. ns, not significant (unpaired two-sided t-test).

d. Lifespan of animals of indicated phenotypes. Young adults were transferred to cold as described in Fig. 1a, incubated for 5 days at 4°C, and then transferred back to 20°C (day 0). Animals were scored every second day and transferred to fresh plates until they stopped laying eggs. After that, nematodes were transferred to fresh plates once or twice a week to avoid lack of food or contamination. The lifespan calculation was made by dividing the number of alive animals by the number of animals at day 0. Scoring continued until all animals were dead. n= 3 independent experiments. At least 100 nematodes were scored per biological replicate.

e. Quantitation of ROS in cold-treated nematodes by dihydroethidium (DHE) staining. Synchronized young adults of the indicated genotypes were incubated at 4°C for 24 h, and ROS were detected after staining with DHE. Changes in DHE fluorescence intensity are expressed relative to wt. Shown are technical replicates (n= 3), and error bars represent SEM. ns, not significant (unpaired two-sided t-test). Note that due to large variation the *p* value between wt and *ets-4* mutant (0.0547) is just above the significance threshold (0.05).

Source data are provided as a Source Data file.

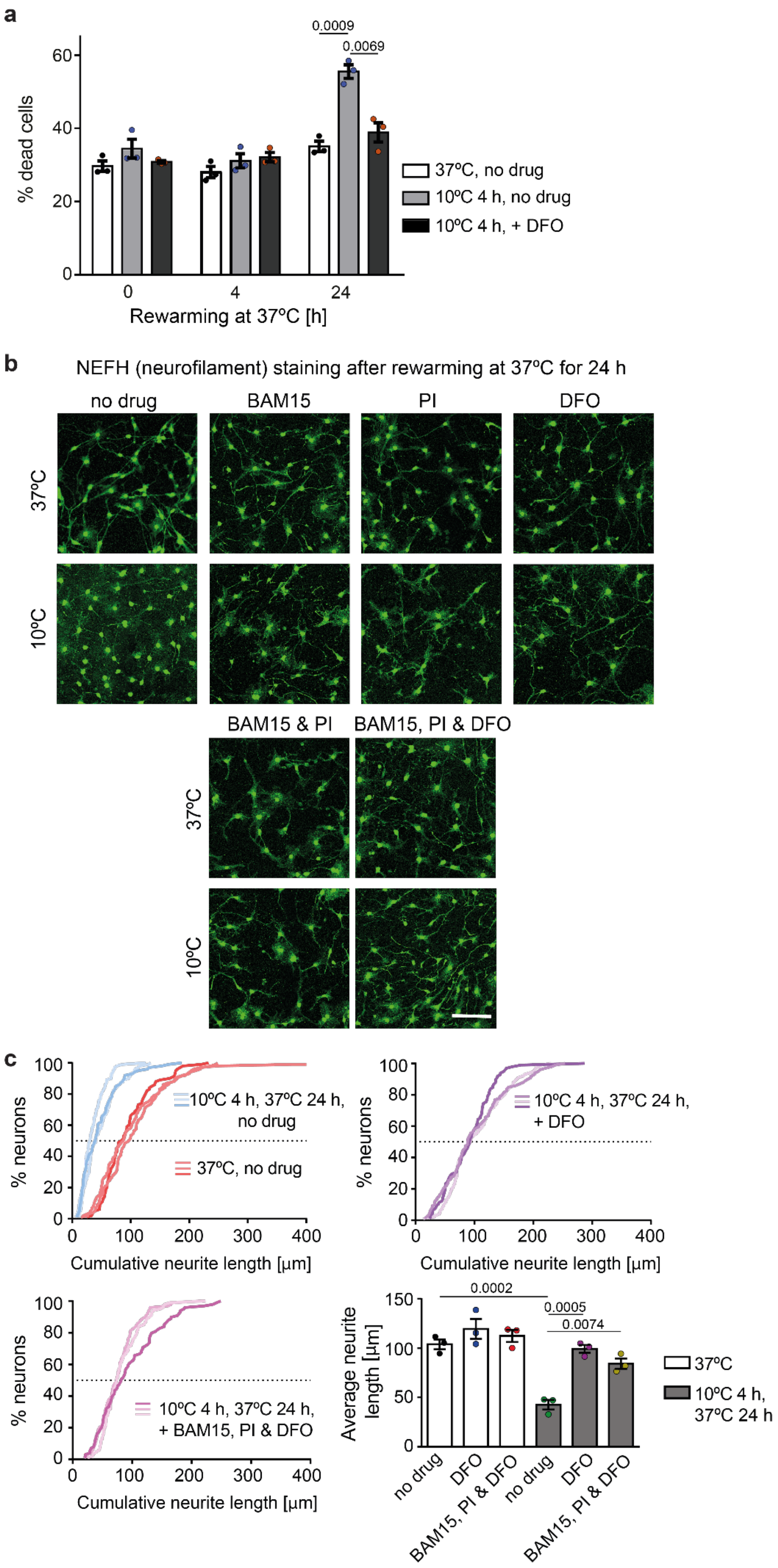
a
% dead cells
Rewarming at 37°C [h]
0.0009
0.0069
37°C, no drug
10°C 4 h, no drug
10°C 4 h, + DFO
b
NEFH (neurofilament) staining after rewarming at 37°C for 24 h
no drug
BAM15
PI
DFO
37°C
10°C
BAM15 & PI
BAM15, PI & DFO
c
% neurons
Cumulative neurite length [µm]
10°C 4 h, 37°C 24 h, no drug
37°C, no drug
10°C 4 h, 37°C 24 h, + DFO
10°C 4 h, 37°C 24 h, + BAM15, PI & DFO
Average neurite length [µm]
0.0002
0.0005
0.0074
no drug
DFO
BAM15, PI & DFO
37°C
10°C 4 h, 37°C 24 h

Figure S6. Lowering iron has a long-lasting protective effect on neural integrity.

Relevant to Fig. 8

a. Deferoxamine rescues murine neurons from death while rewarming. Cells were incubated at 37°C, or subjected to 10°C for 4 h and then rewarmed at 37°C for the indicated time, in the absence or presence of deferoxamine (DFO, 100 μM). Cell viability was examined by staining with propidium iodide. Note that neurons that were dying during rewarming were rescued with DFO. Error bars represent SEM, n= 3 independent experiments. 600-800 cells examined per condition. *p* values calculated using unpaired two-sided t-test.

b. Deferoxamine, similar to BAM15 and protease inhibitors, prevents cold-induced neurite degeneration during rewarming. Differentiated murine neurons were incubated at 37°C, or subjected to 10°C for 4 h and then rewarmed at 37°C for 24 h, in the absence and presence of deferoxamine (DFO, 100 μM), mitochondrial uncoupler BAM15 (100 nM), protease inhibitors cocktail (PI, 1:500 dilution), or their combinations. Shown are representative confocal images taken after staining cells with NEFH to visualize neurofilaments. Note that, in contrast to Fig. 8b, the cells were examined after 24 h rewarming. The neurites appeared well preserved, arguing that the protective effects of drugs are long-lasting. Scale bar: 40 μm.

c. Quantifications of neurite lengths corresponding to **b**. The cumulative plots compare total neurite lengths of differentially treated cells (as indicated). Each curve corresponds to one experimental replicate. The bar graph (bottom right) compares average neurite lengths. Error bars indicate SEM, n= 3 experiments from independently differentiated neuron groups. *p* values calculated using 1-way ANOVA plus post hoc Tukey test.

Source data are provided as a Source Data file.

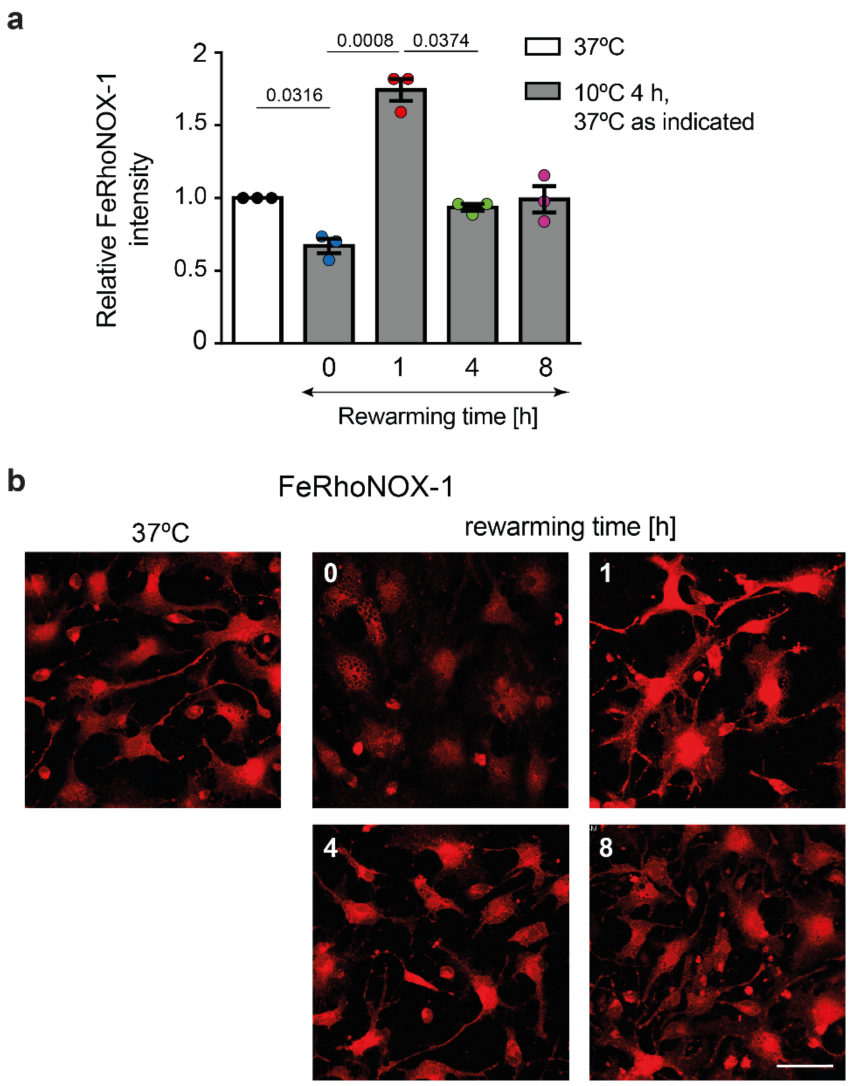

Figure S7. Iron(II) is transiently increased during rewarming of neurons exposed to cold.

Relevant to Fig. 9

a. Iron(II) increases in cold-treated neurons shortly after rewarming. Cells were incubated at 37°C, or subjected to 10°C for 4 h and then rewarmed at 37°C for indicated times. Iron(II) was detected after staining of cells with FeRhoNox-1, and quantified relative to cells incubated at 37°C. Note that iron(II) peaks around 1 h of rewarming. Error bars represent SEM, n= 3 independent experiments. 75-100 cells examined per condition. *p* values calculated using 1-way ANOVA plus post hoc Tukey test.

b. Representative cell images, corresponding to **a**. Note the increased FeRhoNOX-1 fluorescence after 1 h rewarming. Scale bar: 50 µm (same for all panels).

Source data are provided as a Source Data file.

Supplementary Table 1. The *C. elegans* strains used in this work.

Genotype	CGC/RAF/Other
wild type	**N2 (Bristol)**
age-1(hx546) II.; ets-4(rrr16) X.	2169
age-1(hx546) II.	**TJ1052**/1891
daf-16(mu86) I.; age-1(hx546) II.	2150
daf-16(syb707) I.	5010
daf-16(mu86) I.	**CF1038**/1660
ets-4(rrr16) X.	1758
daf-16(mu86) I.; ets-4(rrr16) X.	2107
pqm-1(ok485) II.; ets-4(rrr16) X.	2106
ftn-1(ok3625) V.	**RB2603**/2162
pqm-1(ok485) II.	**RB711**/2104
daf-16(mu86) I.; pqm-1(ok485) II.	2105
pqm-1(ok485) II.; ets-4(rrr16) X.	2033
pqm-1(syb432) II.	2156
pqm-1(syb432) II.; ets-4(rrr16) X.	2157
rege-1(rrr13) I.	5018
rege-1(rrr13) I.; ets-4(rrr16) X.	1759
daf-16(syb707) I.; ets-4(rrr16) X.	5054
daf-16(mu86) I.; pqm-1(ok485) II.; ets-4(rrr16) X.	5062
ftn-1(ok3625) V.; ets-4(rrr16) X.	5063
daf-2(e1370) III.	**CB1370**/1173
daf-2(e1370) III.; ets-4(rrr16) X.	5096
sybSi67[Pdpy-30::ftn-1::unc-54 3'UTR] II.; unc-119(ed3) III.	5069/**PHX1798**
sybSi72[Pvit-5::ftn-1::unc-54 3'UTR] II.; unc-119(ed3) III.	5071/**PHX1920**

ftn-2(ok404) I.	**RB668**/5085
ftn-2(ok404) I.; ets-4(rrr16) X.	5093
ftn-1(syb2550) V.	5118
ftn-1(syb2550) V.; ets-4(rrr16) X.	5112
wuIs57[pPD95.77 sod-5::GFP, rol-6(su1006)]	5106/**GA411**
ftn-1(syb4641) V.	2213
ftn-1(syb4641) V.; ets-4(rrr16) X.	2214

“CGC” indicates strain numbers deposited in the Caenorhabditis Genetics Center; “RAF” strain numbers in the Ciosk lab collection; "Other" strains obtained from other sources.

Supplementary Table 2. Primers for cloning pET28a-derived plasmids expressing recombinant FTNs.

Relevant to Fig. 6

Construct name	Source of ORF	Cloning primers	Mutagenic primers
**pET28a-FTN-1**	*C. elegans* cDNA	fwd:GGAGATATACCATGTCTCTAGCTCGTCAAAACTAT, rev:CTCGAGTGCGGCCGCTTAATCAGAAAATTCCTCTTTGTCGAAC	
**pET28a-FTN-1-E58K/H61G**	pET28a-FTN1	fwd:GGAGATATACCATGTCTCTAGCTCGTCAAAACTAT, rev:CTCGAGTGCGGCCGCTTAATCAGAAAATTCCTCTTTGTCGAAC	fwd:GAGAAGCGTGGCGGTGCCACAGAGCTCATGAG, rev:GCACCGCCACGCTTCTCATCCGATTGCTCCTTGA
**pET28a-FTN-2**	*C. elegans* cDNA	fwd:GGAGATATACCATGTCTCTCGCTCGTCAAAACTAC, rev:CTCGAGTGCGGCCGCTTAATCGGAAAAGTGTTCCTTATCGAAG	
**pET28a-FTN-2-E58K/H61G**	pET28a-FTN2	fwd:GGAGATATACCATGTCTCTCGCTCGTCAAAACTAC, rev:CTCGAGTGCGGCCGCTTAATCGGAAAAGTGTTCCTTATCGAAG	fwd:GAGAAGCGTGAGGGTGCTACCGAGCTCATGC, rev:GCACCCTCACGCTTCTCATCCGATTGTTCCTTGAAG

Supplementary References

1 Heinz, S. *et al.* Simple combinations of lineage-determining transcription factors prime cis-regulatory elements required for macrophage and B cell identities. *Mol Cell* **38**, 576-589, doi:10.1016/j.molcel.2010.05.004 (2010).

2 Tepper, R. G. *et al.* PQM-1 complements DAF-16 as a key transcriptional regulator of DAF-2-mediated development and longevity. *Cell* **154**, 676-690, doi:10.1016/j.cell.2013.07.006 (2013).

3 Sievers, F. *et al.* Fast, scalable generation of high-quality protein multiple sequence alignments using Clustal Omega. *Mol Syst Biol* **7**, 539, doi:10.1038/msb.2011.75 (2011).

4 Kelley, L. A., Mezulis, S., Yates, C. M., Wass, M. N. & Sternberg, M. J. The Phyre2 web portal for protein modeling, prediction and analysis. *Nat Protoc* **10**, 845-858, doi:10.1038/nprot.2015.053 (2015).

The uncropped scan of gels shown in Figure S4d:

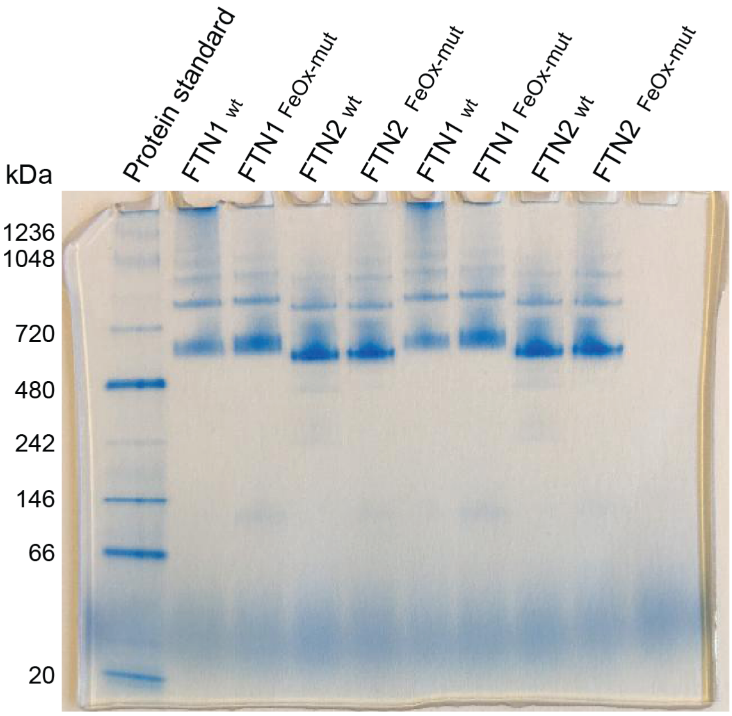

Native-PAGE
24/06/21

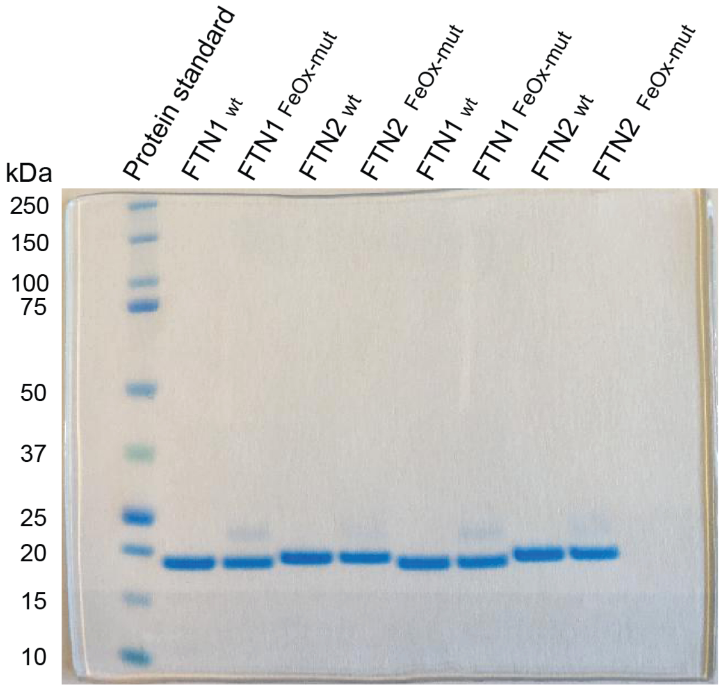

SDS-PAGE
24/06/21
