# Supplementary material for: Ferritin-mediated iron detoxification promotes hypothermia survival in Caenorhabditis elegans and murine neurons
Source: Nat Commun. 2022 Aug 19;13:4883. doi: 10.1038/s41467-022-32500-z (PMC9391379; doi:10.1038/s41467-022-32500-z)
Supplement: Supplementary file 3 — Description of Additional Supplementary Files [file 41467_2022_32500_MOESM3_ESM.pdf]

Description of Additional Supplementary Files

File Name: Supplementary Data 1
Description: Gene expression changes for all detected genes by RNAseq.
The log2 fold changes were calculated based on the average of two biological replicates. Relevant to Fig. 4a.

File Name: Supplementary Data 2
Description: Data and analysis of the *C. elegans* cold-survival experiments. Relevant to multiple figures.
