# Supplementary material for: Ferritin-mediated iron detoxification promotes hypothermia survival in Caenorhabditis elegans and murine neurons
Source: Nat Commun. 2022 Aug 19;13:4883. doi: 10.1038/s41467-022-32500-z (PMC9391379; doi:10.1038/s41467-022-32500-z)
Supplement: Supplementary file 6 — Reporting Summary [file 41467_2022_32500_MOESM6_ESM.pdf]

Corresponding author(s): Rafal Ciosk
Last updated by author(s): Jun 25, 2022

Reporting Summary

Nature Research wishes to improve the reproducibility of the work that we publish. This form provides structure for consistency and transparency in reporting. For further information on Nature Research policies, see our Editorial Policies and the Editorial Policy Checklist.

Statistics

For all statistical analyses, confirm that the following items are present in the figure legend, table legend, main text, or Methods section.

n/a	Confirmed	
☐	☒	The exact sample size (*n*) for each experimental group/condition, given as a discrete number and unit of measurement
☐	☒	A statement on whether measurements were taken from distinct samples or whether the same sample was measured repeatedly
☐	☒	The statistical test(s) used AND whether they are one- or two-sided<br>*Only common tests should be described solely by name; describe more complex techniques in the Methods section.*
☒	☐	A description of all covariates tested
☐	☒	A description of any assumptions or corrections, such as tests of normality and adjustment for multiple comparisons
☐	☒	A full description of the statistical parameters including central tendency (e.g. means) or other basic estimates (e.g. regression coefficient) AND variation (e.g. standard deviation) or associated estimates of uncertainty (e.g. confidence intervals)
☐	☒	For null hypothesis testing, the test statistic (e.g. *F*, *t*, *r*) with confidence intervals, effect sizes, degrees of freedom and *P* value noted<br>*Give P values as exact values whenever suitable.*
☒	☐	For Bayesian analysis, information on the choice of priors and Markov chain Monte Carlo settings
☒	☐	For hierarchical and complex designs, identification of the appropriate level for tests and full reporting of outcomes
☒	☐	Estimates of effect sizes (e.g. Cohen's *d*, Pearson's *r*), indicating how they were calculated

Our web collection on statistics for biologists contains articles on many of the points above.

Software and code

Policy information about availability of computer code

Data collection	No custom software were used in data collection. Poly-A mRNA was sequenced using a Hiseq 50-cycle single-end reads protocol on a HiSeq 2500 device (Illumina). Imaging of C. elegans stained with Oil Red O was performed using either wide-field microscope Z1 (Carl Zeiss) with 10x objective, color camera AxioCam MRc (Carl Zeiss) and using Zen 2.5 (blue edition) software (Carl Zeiss), or using Nikon SMZ25 with DeltaPix color camera with 60x zoom and using DeltaPix inSight Basic Software. Fluorescent imaging of GFP- or mCHERRY-tagged C. elegans nuclei was performed with Carl Zeiss Axio Imager Z2 equipped with a Yokogawa CSU-W1 scan-head, 2 PCO Edge cameras and Plan-Apochromat 40x/1.3 oil objective, and ZEN 2.5 (blue edition) software (Carl Zeiss). Imaging of SOD-5::GFP-expressing C. elegans was performed with Axio Imager.Z2 (Carl Zeiss) equipped with Axiocam 506 mono digital camera (Carl Zeiss) and Plan-Apochromat 63x/1.40 Oil DIC M27 objective, and ZEN 2.5 (blue edition) software (Carl Zeiss). Fluorescence intensity of DHE-stained C. elegans was measured using a Hidex Sense microplate reader. qRT-PCR was performed using a CFXConnect Real-Time System (Bio-Rad, USA) and LightCycler480 (Roche, Switzerland). Imaging of primary neurons stained with various metods was performed using either fluorescence microscope (Leica DMI 4000B, Germany) or confocal fluorescence microscope (Leica TCS SP5, Germany), and LAS X SP8 software. Time-Resolved Measurement (TRM) software (Shimazu, Japan) for LC–ICP-MS was used for controlling both ICP and LC analytical systems, as well as for data collection. Protein MS spectra were aquired with TimsTOF Pro mass spectrometer (Bruker Daltonics, Bremen, Germany).
Data analysis	The Phyre2 web portal was applied to structural alignment of H. sapiens ferritin heavy chain 1 and C. elegans FTN-1, and the Clustal Omega website (https://www.ebi.ac.uk/Tools/msa/clustalo/) or JalView 2.11.2.2 interface (http://www.jalview.org/) for amino acid sequence alignments. Statistical comparison of survival curves between C. elegans strains was done using pairwise Wilcoxon signed rank test, in R v4.0.1. FASTQC was used to check the quality of the raw sequence data and STAR 2.5.0a for C. elegans genome mapping, and EnrichedHeatmap v1.27.0 (an R/Bioconductor package) was used for mRNAseq data analysis and visualization. The sequence motif enrichment analysis was performed with HOMER v4.10, for each cluster with the following parameters: -start -1500, -end 1500, -p 6, and all genes in the genome were used as background. Fiji ImageJ v1.53f51 (public domain software from the National Institutes of Health; http://imagej.nih.gov/ij/) was used for basic image processing, including quantification of mean fluorescent intensity, Oil Red O staining and FeRhoNox-1 staining. In addition, 'Simple Neurite Tracer' plugin in ImageJ was applied to analyze neurite paths. GraphPad Prism v6.01 and v8 were used for statistical analysis and R for statistical comparisons of survival curves and lifespan between strains. Adobe Illustrator v25.0.1

was used for the graphic design of the figures. TRM software (Shimazu, Japan) was used for iron quantification after sample separation (SEC-ICP-MS) by calculating the peak area in the chromatograms, as well as for determining the total iron concentration by direct sample injection (LC-ICP-MS) and quantification based on iron standard solutions. Protein MS data were analyzed with PEAKS Studio v10.6 software.

For manuscripts utilizing custom algorithms or software that are central to the research but not yet described in published literature, software must be made available to editors and reviewers. We strongly encourage code deposition in a community repository (e.g. GitHub). See the Nature Research guidelines for submitting code & software for further information.

Data

Policy information about availability of data

All manuscripts must include a data availability statement. This statement should provide the following information, where applicable:

- Accession codes, unique identifiers, or web links for publicly available datasets
- A list of figures that have associated raw data
- A description of any restrictions on data availability

Source data are provided with this paper. The RNA sequencing data generated during the current study are available in the GEO repository, with the accession No. GSE131870: (https://www.ncbi.nlm.nih.gov/geo/query/acc.cgi?acc=GSE131870). The mass spectrometry proteomics data have been deposited to the ProteomeXchange Consortium via the PRIDE partner repository with the dataset identifier PXD034794 and 10.6019/PXD034794 (https://www.ebi.ac.uk/pride/archive/projects/PXD034794/private). The rest of data supporting the findings of this study are available from the corresponding author upon request.

Field-specific reporting

Please select the one below that is the best fit for your research. If you are not sure, read the appropriate sections before making your selection.

☒ Life sciences ☐ Behavioural & social sciences ☐ Ecological, evolutionary & environmental sciences

For a reference copy of the document with all sections, see nature.com/documents/nr-reporting-summary-flat.pdf

Life sciences study design

All studies must disclose on these points even when the disclosure is negative.

Sample size

Sample sizes were chosen based on previous experience.
In RT-qPCR analysis RNA concentration was determined based on UV absorbance, the same amount of RNA was used to prepare cDNA, and the same cDNA concentration was used to perform RT-qPCR analysis. Around 1000 nematodes were collected per condition to prepare lysate for RNA isolation for polyA mRNA sequencing and RT-qPCR analysis. Equal amounts of RNA were used for further processing as indicated in Methods. The sample size of around 1000 nematodes per condition allows to obtain sufficient amount of total RNA for all further analyses. Moreover, such sample size per dish is optimal to avoid nematode overcrowding and starvation.
Around 100 nematodes were selected for each time point, in each biological replicate, for life curve and lifespan assays, which is optimal to avoid overcrowding and starvation, but provides significant differences between tested samples.
Worms extracts for ICP-MS were diluted to the same concentration based on UV absorbance. The sample size was chosen to obtain iron content value in the standard curve range. The number of animals used for each analysis is delineated within either each respective figure legend or in the further information in the Methods.
During the process of neuronal cell spheres acquisition heads from approx. 10 fetal mice were isolated to establish single-cell suspension culture. Several mice were selected for breeding, based on availability for a given genotype and capability for reproduction. All mice became pregnant, and all available fetuses were collected to generate a large supply of neurospheres. Three separately differentiated cultures of neuronal spheres towards noradrenergic neurons were used to prepare three distinct samples for each experimental group and conduct cell-oriented tests. The variability between estimates was sufficiently small to provide significant differences between test samples in presented studies.
No statistical test was used a priori to determine the sample size. The sample size for C. elegans imaging and cell imaging is enough to obtain a representative group of images.

Data exclusions

No data were excluded from ICP-MS results analyses, cold survival assay, lifespan assay, RT-qPCR results analysis, Oil red O staining analysis and fluorescent imaging results. No data were excluded from protein MS results.
Data from concentration optimization of antioxidant NAC administered to the NGM plates for C. elegans cold survival measurement in the presence of NAC were not included in the manuscript.
Data related to the breeding of female C57BL / 6 mice and their preparation for fetal isolation were omitted.
Data from concentration optimization of antioxidants including DFO, Edaravone, NAC, TEMPOL as well as conducted tests for successful mouse Fth-1 overexpression in neurons after lentiviral transduction were not included in this manuscript. Furthermore collected material from assesing cell quality that is neuronal progenitor markers of neural spheres as well as neronal markers for differentiated noradrenergic neurons were also excluded.

Replication

For ICP-MS experiments, replicates were made using worm extracts prepared from worms synchronized, grown and lysed separetely on different days. For cold survival assay, lifespan assay, RT-qPCR analysis, Oil red O staining analysis and fluorescent imaging biological replicates were prepared by making chunks from separate plates and/or bleaching on separate times/days. Worms were synchronized, grown and analyzed in different times/days. The number of biological replicates of experiments on C. elegans and cells are described in the figure legends.
In vitro experiments with recombinant FTNs were done in technical replicates, and in biologically independent experiments that were performed on different days, using daily-fresh solutions of ferrous ammonium sulfate. Samples for protein MS analysis were prepared in three biological replicates from worm pellets grown and treated on different days. Gel electrophoresis of FTN protein variants were performed twice, each time in two technical replicates, all with similar results.
All procedures, chemicals, compounds and conducted analysis applied to the experiments on neural spheres and differentiated neurons were

standardized as best as possible for reliable, at least triple, replication.
Biological replicates were successfully obtained, except for Figs. 9F and S5E, where only technical triplicates are given, which is clearly indicated in the figures legend.
All replication results are included in the final data.

Randomization

Worms were synchronized and counted: ~1000 L1 larvae for RT-qPCR experiments, ~100 L1 larvae per plate for cold survival assay and lifespan assay, ~1000 L1 larvae were transferred to each NGM plate with OP50 bacteria. For RNAi experiments, worms were synchronized and counted: ~100 L1 larvae were plated onto agar plates containing carbenicillin and IPTG seeded with overnight culture of RNAi bacteria.
The noradrenergic neurons used were differentiated independently and at separate time points.
C. elegans and cells were allocated into experimental groups at random. All data were uniformly collected between the control and assessment groups.

Blinding

ICP-MS data analysis was performed without knowledge about the sample identity. For the rest experiments made on C. elegans blinding was not possible, since it was crucial to know which strain was used in a particular experiment in order to optimize the time of worm transfer to NGM plates, as various strains may differ in development.
Experiments with primary neurons were not blinded because the settings for acquisition of fluorescence signals had to be adjusted for the control samples in order to avoid signal oversaturation in the remaining samples. The fluorescence microscopy images were then taken using identical settings for all samples, therefore the blinding was irrelevant for the interpretation of results.

Reporting for specific materials, systems and methods

We require information from authors about some types of materials, experimental systems and methods used in many studies. Here, indicate whether each material, system or method listed is relevant to your study. If you are not sure if a list item applies to your research, read the appropriate section before selecting a response.

Materials & experimental systems

n/a	Involved in the study
☐	☒ Antibodies
☐	☒ Eukaryotic cell lines
☒	☐ Palaeontology and archaeology
☐	☒ Animals and other organisms
☒	☐ Human research participants
☒	☐ Clinical data
☒	☐ Dual use research of concern

Methods

n/a	Involved in the study
☒	☐ ChIP-seq
☒	☐ Flow cytometry
☒	☐ MRI-based neuroimaging

Antibodies

Antibodies used

The antibodies utilized herein are as follows:
Nestin (mouse anti-nestin; 1: 500; DSHB, USA; Rat-401-s),
Foxg-1 (rat anti-FoxG1; 1:100; Abcam, UK; ab18259; LOT: GR30760-1),
Emx1 (rat anti-Emx1; 1: 100; Millipore, USA; ab15067; LOT: NG1554210)
Emx2 (rabbit anti Emx2; 1:100; Abgent, USA; AP5542c; LOT: SA100408AF)
Th (mouse anti-TH; 1:100; Abcam, UK; ab112; LOT: GR3204690-7),
S100b (rat anti-S100B; 1:100; Abcam, UK; ab52642; LOT: GR3215095-1),
DβH (sheep anti-DBH; 1:500; Abcam, UK; ab19353; LOT: GR287731-12),
Darpp32 (rat anti-DARPP32; 1:50; Abcam, UK; ab40801; LOT: GR286695-12),
NEFH (mouse anti-NEFH;1:50; DSHB, USA; RT97).

Validation

Except for nestin and NEFH, all remaining antibodies were used only to check the progress of neurosphere's differentiation. All antibodies are commercially available, and have been validated by their corresponding manufacturer's.
Anti-Foxg1 (ab18259) – validated for western blot and immunofluorescence by manufacturer. Reacts with mouse, rat and human endogenous levels of total FOXG1 protein.
Anti-Emx1 (ab15067) – validations by manufacturer are no available. Reacts with human endogenous levels of total EMX1 protein.
Anti-Emx2 (AP5542c) - validated for western blot by manufacturer. Reacts with human endogenous levels of total EMX2 protein.
Anti-Th (ab112) – validated for western blot and immunohistochemistry by manufacturer. Reacts with rat endogenous levels of TH protein.
Anti-S100b (ab52642) – validated for western blot and immunofluorescence by manufacturer. Reacts with mouse, rat and human endogenous levels of total S100b protein.
Anti-DBH (ab19353) – validated for immunofluorescence by manufacturer. Reacts with rat endogenous levels of DBH protein.
Anti-Darpp32 (ab40801) – validated for western blot and immunofluorescence by manufacturer. Reacts with mouse, rat and human endogenous levels of total Darpp32 protein.

Eukaryotic cell lines

Policy information about cell lines

Cell line source(s)	Primary neuronal stem cells as neural progenitor spheres were established using fetal C57BL/6 mouse strain. HEK293T cell line was purchased from ATCC (no. CRL-3216™, ATCC, Virginia, USA).
Authentication	Neural progenitor spheres were checked for the presence of protein markers on neuronal progenitors by the authors of the article. HEK293T cells have been authenticated by ATCC company at source.
Mycoplasma contamination	Cell cultures were not tested for Mycoplasma contamination.
Commonly misidentified lines (See ICLAC register)	No commonly misidentified lines were used in the study.

Animals and other organisms

Policy information about studies involving animals; ARRIVE guidelines recommended for reporting animal research

Laboratory animals	In this study we used a hermaphroditic species, Caenorhabditis elegans, strains: The strains used in this work: wild type (N2, Bristol); RAF2169 age-1(hx546) II.; ets-4(rrr16) X., TJ1052 age-1(hx546) II., RAF2150 daf-16(mu86) I.; age-1(hx546) II., RAF5010 daf-16(syb707) I., CF1038 daf-16(mu86) I., RAF1758 ets-4(rrr16) X., RAF2107 daf-16(mu86) I.; ets-4(rrr16) X., RAF2106 pqm-1(ok485) II.; ets-4(rrr16) X., RB2603 ftn-1(ok3625) V., RB711 pqm-1(ok485) II., RAF2105 daf-16(mu86) I.; pqm-1(ok485) II., RAF2033 pqm-1(ok485) II.; ets-4(rrr16) X., RAF2156 pqm-1(syb432) II., RAF2157 pqm-1(syb432) II.; ets-4(rrr16) X., RAF5018 rege-1(rrr13) I., RAF1759 rege-1(rrr13) I.; ets-4(rrr16) X., RAF5054 daf-16(syb707) I.; ets-4(rrr16) X., RAF5062 daf-16(mu86) I.; pqm-1(ok485) II.; ets-4(rrr16) X., RAF5063 ftn-1(ok3625) V.; ets-4(rrr16) X., CB1370 daf-2(e1370) III., RAF5096 daf-2(e1370) III.; ets-4(rrr16) X., PHX1798 sybSi67[Pdpy-30::ftn-1::unc-54 3'UTR] II.;unc-119(ed3) III., PHX1920 sybSi72[Pvit-5::ftn-1::unc-54 3'UTR] II.;unc-119(ed3) III., RB668 ftn-2(ok404) I., RAF5093 ftn-2(ok404) I.; ets-4(rrr16) X., RAF5118 ftn-1(syb2550) V., RAF5112 ftn-1(syb2550) V.;ets-4(rrr16) X., GA411 wuIs57[pPD95.77 sod-5::GFP, rol-6(su1006)], RAF2213 ftn-1(syb4641) V., RAF2214 ftn-1(syb4641) V.; ets-4(rrr16) X.<br>The sex of the mice fetuses was not checked. They were taken for isolation at gestation day between E9-11.<br>The animals in the experiment were kept in constant, strictly defined, regulated and controlled atmospheric conditions, i.e. at a temperature of 22°C +/- 2°C, air humidity 55-60%, in a daily cycle of 12 hours of light (12 hours continuous) / 12 hours of darkness (continuity of 12 hours), in the experimental and living room, where the air exchange is at the level of 8-15 changes / h.
Wild animals	Study did not involve wild animals.
Field-collected samples	Study did not involve samples collected from the field.
Ethics oversight	Studies on C. elegans require no ethical approval.<br>Pregnant female mice were used as source of fetal brain tissues to derive primary differentiated neurons of mammalian origin. Collection of mouse embryonic brain tissues was approved by the Local Ethics Committee at Poznan University of Life Sciences, Poland.

Note that full information on the approval of the study protocol must also be provided in the manuscript.
